# Is African Swine Fever Driven by Flying Hematophagous Insects?

**DOI:** 10.3390/pathogens14060563

**Published:** 2025-06-05

**Authors:** Marek Walczak, Maciej Frant, Krzesimir Szymankiewicz, Małgorzata Juszkiewicz, Katarzyna Podgórska, Marcin Smreczak

**Affiliations:** Department of Virology and Viral Animal Diseases, National Veterinary Research Institute, Partyzantow 57, 24-100 Pulawy, Poland; maciej.frant@piwet.pulawy.pl (M.F.); krzesimir.szymankiewicz@piwet.pulawy.pl (K.S.); malgorzata.juszkiewicz@piwet.pulawy.pl (M.J.); katarzyna.podgorska@piwet.pulawy.pl (K.P.); marcin.smreczak@piwet.pulawy.pl (M.S.)

**Keywords:** insects, indirect transmission, ASF, ASFV

## Abstract

African swine fever (ASF) has become one of the most economically important diseases affecting swine and has a significant negative impact on the global pork production sector. In Europe, the main reservoir of the disease is the wild boar population, which poses a risk of transmitting the disease to pig farms. To date, no safe and effective vaccine is available on the market. Therefore, biosecurity measures and early recognition of the disease play a key role in preventing and combating ASF. In recent years, numerous insights into the nature of the virus have emerged; however, several knowledge gaps still need to be addressed. One of these gaps is an accurate understanding of all possible pathways through which the virus can reach a pig farm. Interrupting these pathways would significantly reduce the risk of disease outbreaks. Despite a general understanding of disease transmission, ASF can still affect farms with well-established high biosecurity measures. This article highlights the potential for mechanical transmission of ASF by flying hematophagous insects, considering several factors, including current knowledge of the putative role of insects in ASF transmission, insects’ abilities to transmit the virus, ASFV properties, the uncertainties regarding the effectiveness of indirect transmission, and the seasonality of disease outbreaks on domestic pig farms.

## 1. Introduction

African swine fever virus (ASFV) is a causative agent of African swine fever (ASF). Since 2007, the disease has been continuously spreading across Europe and Asia, paralyzing the world’s pork production sector by affecting domestic pigs. The mortality due to the disease is high and may reach 100% in the affected animals, as there is no safe and effective vaccine available in the EU market [1,2]. Therefore, biosecurity measures and early recognition of the disease play a key role in preventing and combating ASF. Despite all efforts, the experience of the ASF epidemic has shown that the disease can affect farms even with a defined high level of biosecurity [3]. Recent years have brought numerous insights into the virus’s characteristics; however, there are still several knowledge gaps that need to be revealed. One of these gaps is the precise understanding of all possible pathways through which the virus can reach a pig farm. Interrupting these pathways would significantly reduce the risk of disease occurrence.

In Europe, the wild boar population is the main reservoir of the ASF virus, and the occurrence of the disease on pig farms is closely linked to the presence of these animals in the environment. The ASF spread rate can vary in nature, primarily depending on the wild boar density. The estimated rate is 1–2 km per month [4,5,6]. Human activity can accelerate this process, as demonstrated in the case of the Lubuskie Voivodeship in Poland, where the virus was found 300 km from the nearest ASF-affected area [7]. ASF transmission can occur both directly (e.g., wild-boar-to-wild-boar, wild-boar-to-pig, and pig-to-pig contact) and indirectly (through contaminated objects, feed, clothing, etc.). The virus can also be transmitted via aerosol; however, only over short distances (up to 10 m) [8,9], and the virus’s half-life in aerosol has been estimated at approximately 14 min [10]. Therefore, while aerosol transmission is not entirely ruled out, it poses a lower risk compared to other pathways in the process of virus spread from wild boars to pig farms.

An important pathway for ASF transmission in certain regions involves soft ticks [11]. This mode of transmission has been well documented in Africa, where, in the sylvatic cycle, ticks of the *Ornithodoros* genus transmit the virus to susceptible animals belonging to *Suidae*. In contrast to soft ticks, hard ticks (widely present in Europe) have been ruled out as ASF vectors by several authors [12,13]. Nevertheless, in studies conducted in Poland, the infectious virus was isolated from a tick belonging to the species *Dermacentor reticulatus*, which was found on wild boars infected with ASF [14]. While this finding suggests a potential for mechanical transmission, the life cycle of hard ticks (more specifically, their limited ability to switch hosts and limited mobility) poses a low risk of ASF transmission under natural conditions. Due to the aforementioned reasons, the focus of this article was narrowed to a more specific group within Arthropoda phylum, the class of Insecta.

In contrast, the biology of blood-sucking insects allows for repeated feeding on multiple hosts. Moreover, these insects come into contact with cells supporting ASFV replication, which, combined with the ability to travel considerable distances, makes them a potential threat to pig farming. Recent publications regarding the ineffective indirect transmission of ASF [15,16,17,18] suggest that other potential transmission routes to pig farms (beyond the generally accepted ones) should also be explored. Additionally, the observed seasonality of ASF outbreaks in domestic pigs, correlating with the period of heightened insect activity, suggests that insect-vectored transmission remains a possibility.

This article aims to highlight the potential for mechanical transmission of ASF by flying hematophagous insects, considering several factors, including current knowledge of the putative role of insects in ASF transmission: insects’ abilities to transmit the virus, ASFV properties, the uncertainties about the effectiveness of indirect transmission, and the seasonality of disease outbreaks on domestic pig farms.

## 2. Indirect Transmission Pathways of African Swine Fever: Implications for Biosecurity and Disease Control

It is generally accepted that indirect transmission of ASF has to be taken into consideration in the disease control efforts. Actions reducing the risk of ASF spread via contaminated objects, feed, animal-derived materials, or human activity must be undertaken to limit the spread of the virus [19]. Indirect transmission was the cause of the first introduction of ASF into Europe in 1957, when pigs were fed meat containing ASFV [20]. In the case of long-distance spread, human activity is considered a key contributing factor. Such suspicions arose, for example, in the Czech Republic in 2017, Belgium in 2018, and Poland in 2019, when ASF appeared in new areas that were significantly distant from the nearest previously recorded cases [7,21]. However, these incidents have not been definitively explained in terms of how ASF entered wild boar populations. Similarly, in multiple outbreaks, it has not been explained how ASF entered pig farms. Beyond the generally recognized pathways, such as the introduction of ASF-affected animals or non-compliance with biosecurity measures, no detailed information that would allow for a clear identification of the transmission route was available. Recent research has called into question the actual risk posed by indirect transmission. Studies using sentinel pigs have shown that infection through a contaminated environment is not easily achieved. In the study by Olesen [18], infection occurred only within a maximum of one day after the introduction of sentinel pigs into a contaminated pen, or not at all [15]. Similar findings have been reported in other studies, where researchers were unable to infect pigs via environmental exposure or to isolate infectious virus from contaminated environmental samples [16,17]. When evaluating the risk of indirect ASF transmission, several factors must be taken into account. There is a significant difference between introducing meat products, kitchen waste, or similar materials that may contain infectious ASF virus to wild boars or pigs and the incidental contact of fomites with trace amounts of the virus. Special attention should be given to understanding the underlying causes of ASF outbreaks on farms with high biosecurity standards [3], where risk factors such as swill feeding or personnel negligence (e.g., failure to change clothing or footwear or to implement disinfection protocols) have been ruled out. In explaining these phenomena, the missing link could be blood-feeding insects, which—due to their contact with blood and their biological capabilities—might represent a significant and still underestimated risk.

## 3. Seasonality of ASF Outbreaks on Pig Farms

**Figure 1:**
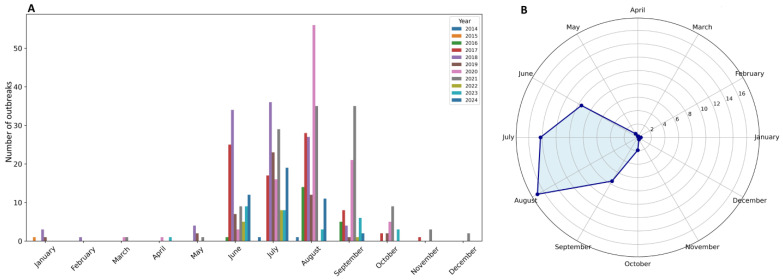
Number of ASF outbreaks on pig farms in Poland from 2014 to 2024, grouped by month (**A**), and the average number of outbreaks per month over the same period (**B**), based on data of the General Veterinary Inspectorate of Poland [24].

The seasonality of ASF outbreaks in pig farms, which occur mainly in summer and early autumn, remains strong indirect evidence for the potential role of insects in ASF transmission. The summer season coincides with insect activity. As noted by Semelbauer et al., the presumed seasonal flight activity of stable flies in southwestern Slovakia exhibits an intermediate pattern, characterized by a major peak in summer followed by a smaller secondary peak in autumn [22]. An increase in outbreaks on domestic pig farms in southeastern Romania has been observed, especially near water bodies and following rainfall, when conditions are more favorable for insects [21]. A similar observation was made by Turčinavičienė et al. in Lithuania [23]. As illustrated in Figure 1A, ASF outbreaks on pig farms in Poland occur predominantly from June to September. The average number of pig outbreaks reaches its peak in August, with an average of 17 outbreaks (Figure 1B), based on data from 2014 to 2024.

Some authors attribute the seasonality of ASF to increased human activities in both domestic pigs’ and wild boars’ environments [25]. Pig farm activities vary by season, with summer posing a higher risk of contact with infected wild boars or contaminated environments. During warmer months, pigs may be fed freshly cut forage, allowed outdoor access, and exposed to increased farm activity. Crop harvesting and frequent movement of farm vehicles and machinery between the farm and potentially contaminated fields further heighten the risk [26]. As indicated by Kim et al., it is unlikely that the summer/autumn peaks could be solely attributed to seasonal changes in pig production. Authors indicated that pig production in Europe and in China did not increase during the summer and autumn [27]. Observed seasonality may suggest the presence of additional factors. The efficiency of indirect transmission via currently acknowledged means may be less efficient than previously recognized, leaving room for insect vectors to conceivably mediate higher transmission rates and thus outbreaks in summer.

## 4. Insects as Vectors of Virus Diseases: The Potential Role of Insects in ASF Transmission

A widely accepted definition characterizes a vector as any organism (vertebrate or invertebrate) that serves as a carrier of an infectious agent, transmitting it between organisms of different species [28]. In the case of hematophagous insects, mechanical transmission of pathogens occurs when the insects are interrupted during blood feeding on an infected host and subsequently move to a susceptible host to complete their blood meal [29]. To date, the transmissions of several pathogens that cause animal diseases by insects have been documented, including viruses, bacteria, and parasites. Major insect-vectored, viral diseases of animals are presented in Table 1 [30,31,32,33,34,35,36,37,38,39].

The role of insects in ASF transmission has also been investigated. In a study by Saegerman et al., experts evaluated ten criteria and identified vectoral competence, abundance, and biting rate as the most important factors facilitating disease transmission. Additionally, stable flies (*Stomoxys calcitrans*) were considered the most probable vector of ASF [40]. In one of its latest reports, the EFSA also highlighted the potential role of stable flies and horse flies, while emphasizing the considerable knowledge gaps that still exist on this topic [41]. In studies conducted by Mellor et al., stable flies were shown to efficiently transmit ASFV to naïve pigs. Mellor fed 30 and 57 flies with blood containing the ASFV (10^8^ HAD_50_/mL) and then allowed them to bite two naïve pigs—either 1 or 24 h later—both of which became infected. Moreover, he demonstrated that after 48 h, the viral titer in the fly’s body had not decreased; however, he was unable to isolate infectious virus on the third day and beyond post-blood feeding [42]. Olesen et al. successfully isolated infectious virus from various parts of *S. calcitrans* within 0–72 h after the flies were artificially fed ASFV-containing blood [43]. These studies have clearly proven that ASFV can be mechanically transmitted by insects of this species. An interesting study was presented by Vergne et al. [44], where the authors employed a mechanistic vector-borne transmission model for ASF virus within an outdoor domestic pig farm to assess the relative contribution of stable flies to the spread of the virus. The study linked the number of insects present to the percentage of transmission cases. The model was adapted to the ecology of the vector, its blood-feeding behaviors, and the dynamics of transmission between pigs. When there are fewer than five flies per pig, stable flies may be responsible for ASF transmission in approximately 10% of cases. However, as the number of insects increases to 20 or 50, this percentage rises to 30% and 50%, respectively. These findings emphasize the importance of insecticidal treatments and consistent insect population management to minimize the risk of the virus transmission.

Regarding other insect species, a study conducted in Estonia examined various groups of blood-feeding arthropods collected during the summer of 2017 in wild boar habitats on the Estonian island of Saaremaa. Saaremaa had the highest prevalence of ASF in Estonia, with an infection rate of 9% among hunted wild boar. Herm et al. analyzed 6274 biting midges, 77 tabanids, 757 mosquitoes, and additionally, 784 ticks, either individually or in pools. No ASFV DNA was detected in any of the samples [45]. However, two years later, Herm et al. confirmed the presence of ASFV DNA in samples of flies and mosquitoes caught at a pig farm affected by the disease [46]. Similar to the findings of the initial study by Herm, Vasić et al. were also unable to detect ASFV DNA in fly samples collected in Serbia [47]. Importantly, as reported by several authors in studies conducted in Lithuania, ASFV genetic material was detected in hematophagous insects collected near ASF-free farms. Turčinavičienė et al. detected ASFV DNA in samples of mosquitoes (*Culex* spp.) and flies (*Lucilia* spp., *Musca domestica*, and *S. calcitrans*) [23]. In 2023, Stedler et al. found ASFV DNA in 1 of 5 tested pools of tabanids but not in fly or mosquito samples [48], while Olesen confirmed the presence of ASFV genetic material in 6/48 pools of flies [49]. These findings suggest that contamination originated from outside the farms (namely, from the wild boar population). However, according to Turčinavičienė, the role of Stomoxys flies as mechanical vectors may be incidental, as no significant correlation was found between S. calcitrans activity and the number of ASF outbreaks in pig farms [23]. Recent studies by Dhollander et al. have shown that ASFV DNA may also be detectable in biting midges and stable flies. In these studies, a total of 8604 biting midges and 742 *Stomoxys calcitrans* flies were collected from selected pig farms in Poland, Romania, and Lithuania. ASFV DNA was detected in 27 out of 1219 insect pools, with positive pools predominantly found in *Culicoides punctatus*, *C. newsteadi*, and the *Obsoletus* complex. Most detections occurred in August. However, attempts to isolate infectious ASFV were unsuccessful [50].

The role of insects in ASF transmission has also been considered in the context of their contact with the carcasses of dead wild boars or pigs. Froth et al. ruled out the involvement of blowflies (*Lucilia sericata* and *Calliphora vicina*) in disease transmission. The larvae and pupae of these insects were experimentally raised on ASFV-infected spleen tissue and tested for infectious virus and viral DNA after various time intervals. Using qPCR, ASFV DNA was detected in the larvae and pupae, even after several washing steps, confirming that the virus was ingested during the larval feeding stage. However, infectious virus was never isolated. The authors suggested that the enzymes present in the larvae inactivated the virus [51]. Similarly to Froth, Olesen demonstrated that the mealworm (*Tenebrio molitor*) and the black soldier fly (*Hermetia illucens*), after ingesting ASFV, were qPCR-positive for up to 3 or 9 days. However, the oral uptake of these insects did not result in infection in naïve pigs [52]. In 2024, Vasić et al. reported that ASFV DNA may be detectable in adult flies (*L. sericata* and *S. calcitrans*) experimentally raised on ASF-infected pig carcasses [47]. The aforementioned findings are summarized in the Table 2.

The phenomenon of detecting ASFV DNA in the insect samples while failing to detect infectious virus is most probably related to the fact that the virus remains infectious in flies for a relatively short period (up to approximately 48 h) [42,43]. The decline in DNA concentration observed by Olesen et al. indicates viral degradation within the insect’s body. These findings align with observations made by Froth et al., who suggested that the virus is inactivated by enzymes present in the digestive system of flies [51]. However, this does not negate the risk of transmission, which is most likely limited to a short period following the ingestion of blood by the insects. As demonstrated by the aforementioned studies, the transmission pathways of the ASFV to susceptible animals via insects should be analyzed independently, with particular focus on hematophagous insects that come into contact with infected blood and have the potential to transmit the virus to subsequent hosts.

Based on the reviewed studies, it is evident that ASFV can remain infectious in hematophagous insects, particularly Stomoxys calcitrans, for up to 48 h after feeding on infected blood. Experimental findings confirm that such flies are capable of transmitting the virus to naïve pigs, highlighting their potential as mechanical vectors under certain conditions. Although field studies frequently detect ASFV DNA in various insect species, infectious virus may not be isolated, suggesting a limited window of vectorial capacity. This discrepancy likely results from rapid viral degradation within the insect’s body. Nevertheless, the demonstrated experimental transmission and repeated detection of viral DNA in field-collected insects underscore the role of hematophagous insects in ASFV epidemiology.

## 5. Biological Parameters Influencing Insect-Mediated ASFV Transmission: Infectious Dose, Route of Infection, Feeding Behavior, and Dispersal

ASFV exhibits a strong tropism to the cells of myeloid lineage (i.e., monocytes and macrophages), which are abundantly present in the bloodstream. During the peak of viremia, the blood of infected animals can contain high concentrations of the virus, approximately 10^8^ hemadsorbing units (HAU) per milliliter of blood [55]. Consequently, blood represents one of the materials with the highest biological potential for ASFV transmission.

The route of infection is also crucial. Ingestion of infected insects as a transmission route cannot be ruled out, as demonstrated by Olesen et al. [53]. However, the risk of transmission via ingesting ASFV-positive insects is considered lower than risk of transmission through bites of ASFV-positive insects, which conceivably inject the virus directly into the host’s bloodstream. The oral route requires a significantly higher dose [56], whereas intramuscular or intranasal infection can induce disease in susceptible animals with significantly lower viral doses (i.e., 3–10 HAU) [57,58]. In stable flies, ASFV DNA is predominantly detected in the head and body regions following a blood meal, with lower concentrations in the mouthparts [43]. Stable flies can consume an average of 11–15 μL of blood per meal [59] and may regurgitate ingested blood if they ingest more than they can digest. *S. calcitrans* can partially regurgitate up to 2 µL of blood or digest it in the midgut after storing it in the crop for over 24 h [60]. The amount of regurgitated blood seems to be sufficient for effective host infection, even in cases of moderate viremia in the animals on which the insects previously fed. However, studies presented by Olesen, Mellor, and Vergne indicated that a single stable fly may not be sufficient to establish infection via biting, and the number of stable flies present may play a crucial role in the effective transmission of ASF [42,43,44]. In Tabanids, the blood-meal size of a female ranges from 20 μL in smaller species to up to 600 μL in larger species [61]. In mosquito species, blood feeding is also primarily restricted to females, with blood meals reaching up to 10 µL depending on the species [60,62]. Regarding biting midges, adult females consume approximately 0.1 µL of blood every three to four days [60]. This suggests that a greater number of insects and multiple bites may be necessary for effective virus transmission.

It is significant that some insects can traverse the necessary distance between potential reservoirs and pig farms and bite hosts multiple times. The active flight range of *Stomoxys calcitrans* varies from approximately 1.5 to 5.0 km [60,63], while wind dispersal can extend up to 13 km [63]. The flight range of *Culicoides* is limited to a few hundred meters; however, they can be passively dispersed over long distances by wind, further influencing their role in disease epidemiology [64,65,66]. Most mosquitoes have limited dispersal ability through active flight; however, flight distances of 2–3 km have been reported in some *Aedes*, *Culex*, and *Anopheles* species [67]. The situation is different for Tabanids, which have a limited flight range of approximately 200 m, restricting their ability to serve as efficient mechanical vectors over long distances. While large groups of hosts may attract Tabanids from farther away due to their strong olfactory signature, individual hosts beyond this range are less likely to contribute to transmission. This constraint makes mechanical transmission an occasional and highly variable phenomenon, dependent on specific ecological factors such as vector density and host distribution [61]. The most important abilities of insects in terms of ASF transmission are summarized in Table 3.

As demonstrated by the aforementioned factors, potential ASFV transmission through insects may be influenced by several key determinants, including the viral load in the blood, the feeding behavior of the insects, and their flight range. Blood-feeding insects, such as stable flies, mosquitoes, and biting midges, are particularly important as vectors due to their ability to directly inject the virus into the host’s bloodstream while feeding. The risk of ASFV transmission increases with the number of insect bites and the density of insect populations. Insects that feed multiple times on infected hosts have a higher likelihood of acquiring and transmitting the virus. The risk is particularly high when larger numbers of insects are involved in feeding on infected animals, as this increases the chances of the virus being spread to new hosts. Insects with extended flight ranges, such as stable flies and mosquitoes, have the potential to disperse ASFV over considerable distances, thereby increasing the risk of transmission across farms and regions. Consequently, controlling insect populations and minimizing their capacity to transmit the virus through targeted biosecurity measures is crucial in preventing the further spread of ASFV.

## 6. Conclusions

The ASF epidemic, which has been developing in Europe for nearly 20 years, demonstrates how difficult it is to combat this disease. Given natural conditions such as the density of the wild boar population, ASF is likely to remain active in the environment for a long time. Therefore, efforts should primarily focus on establishing pig farms as secure enclaves, free from ASF. To achieve this, all potential risk factors must be considered—starting with a detailed analysis of the local epidemiological situation, through wild boar population control, and ending with comprehensive biosecurity measures that minimize the risk of the disease reaching farm premises. To address the potential risk of ASF transmission by insects, biosecurity measures should include insecticide treatments, insecticidal lamps, nets, traps, and filters in ventilation systems to reduce insect populations. Proper fencing can reduce the risk by increasing the distance from wild boar habitats and agricultural crops which attract wild boars, thus increasing the distance between the potential vector and the host.

To date, there is no conclusive evidence in the literature confirming the role of insects as mechanical vectors of ASF transmission. However, the EFSA and multiple authors of studies in this field have seriously considered this risk. While the role of insects in ASF transmission is not yet definitively proven, various indirect evidence and observations support their potential involvement:

1. Detection of infectious virus in the insects and/or efficient transmission to pigs under experimental conditions;
2. Detection of ASFV DNA in insect organisms collected from ASF-free farms;
3. Myeloid cells, predilected for ASF replication abundant in blood;
4. Blood-feeding insects’ abilities—volume of blood ingested, regurgitation ability, multiple biting behavior, and flight range;
5. Seasonality of ASF outbreaks in pig farms, including those with high biosecurity standards.

The assumption that flying hematophagous insects can play a contributory role in ASFV transmission carries several important implications for disease control. While direct contact remains the primary route of ASF spread, the recognition of this potential indirect pathway highlights the need for refined and enhanced control strategies. Increasing farmers’ and veterinarians’ awareness on this subject and applying targeted insect control should be considered as an important preventive tool.

## Figures and Tables

**Table 1:** Major insect-vectored viral diseases of animals and their primary vectors.

Pathogen Name	Insect Vectors	Host	**References**
West Nile Virus (WNV)	Mosquitoes	Birds, horses, dogs, cats	[30,31,32,33]
Rift Valley Fever (RVFV)		Ruminants	
Equine Encephalitis Virus (Eastern, Western, and Venezuelan)		Horses	
Japanese Encephalitis Virus (JEV)		Pigs, horses, birds	
Bluetongue Virus (BTV)	Biting midges	Ruminants	[34,35,36,37]
Epizootic Hemorrhagic Disease Virus (EHDV)		Ruminants	
African Horse Sickness Virus (AHSV)		Horses, donkeys, zebras	
Schmallenberg Virus (SBV)		Ruminants	
Vesicular Stomatitis Virus (VSV)	Flies	Horses, cattle, pigs	[38,39]
Bovine Leukemia Virus * (BLV)		Cattle	

*—potentially.

**Table 2:** Studies on the potential role of insects in ASFV transmission.

First Author, Year	Sample Species	Infectious Virus Isolation	ASFV DNA Detection	Comment	References
Mellor, 1987	Stable flies	Yes (up to 48 h)	n/p	Experimental study/ efficient ASFV transmission to pigs via biting	[42]
Herm, 2017	Biting midges, tabanids, mosquitoes, ticks	n/p	No	Environmental study/ high-ASF-prevalence area	[45]
Forth, 2018	Blowflies (larvae)	No	Yes	Experimental study/potential contact with WB carcasses	[51]
Olesen, 2018	Stable flies	Yes (0–72 h)	Yes	Experimental study/ efficient ASFV transmission to pigs via ingestion	[43,53]
Herm, 2020	Flies, mosquitoes	n/p	Yes	Environmental study/ ASF-affected pig farm	[46]
Turčinavičienė, 2021	Flies, mosquitoes	n/p	Yes	Environmental study/ ASF-free pig farms	[23]
Olesen, 2022	Mealworm, black soldier fly (larvae)	No	Yes	Experimental study/inefficient transmission via ingestion	[52]
Olesen, 2023	Flies	n/p	Yes	Environmental study/ ASF-free pig farms	[49]
Stelder, 2023	Tabanids	n/p	Yes	Environmental study/ ASF-free pig farms	[48]
Balmoș, 2023	Stable flies, biting midges	n/p	Yes	Environmental study/ ASF-affected pig farm	[54]
Vasić, 2024	Flies	n/p	No (En) Yes (Ex)	Environmental study (En)/ experimental study (Ex) insects raised on pig carcasses	[47]
Dhollander, 2025	Stable flies, biting midges	No	Yes	Environmental study/ ASF-free and ASF-affected pig farms	[50]

n/p—not performed, WB—wild boar.

**Table 3:** Biological traits influencing insect capacity for ASFV transmission.

Family (Common Name)	Active Flight Range (km) *	Blood Meal Volume (µL)	Regurgitation Ability	Multiple Bites Ability	References
Muscidae (*Stable flies*)	1.5–5.0	11–15	Yes	Yes	[59,60,63]
Ceratopogonidae (*Biting midges*)	0.7	0.1	Yes	Yes	[64,65,66]
Culicidae (*Mosquitoes*)	2–3	10	Yes	Yes	[67]
Tabanidae (*Horseflies*, *Deerflies*)	0.2	20–600	Yes	Yes	[60,61]

*—can be extended by wind, km—kilometers.
